# Analyzing Serum-Stimulated Prostate Cancer Cell Lines After Low-Fat, High-Fiber Diet and Exercise Intervention

**DOI:** 10.1093/ecam/nep031

**Published:** 2011-03-15

**Authors:** Sherry Soliman, William J. Aronson, R. James Barnard

**Affiliations:** ^1^Department of Physiological Science, University of California, Los Angeles, CA 90095, USA; ^2^Department of Urology, Geffen School of Medicine, University of California, Los Angeles, CA 90095, USA

## Abstract

Serum from men undergoing a low-fat, high-fiber diet and exercise intervention has previously been shown to decrease growth and increase apoptosis in serum-stimulated, androgen-dependent LNCaP cells associated with a reduction in serum IGF-I. Here we sought to determine the underlying mechanisms for these anticancer effects. Again, the intervention slowed growth and increased apoptosis in LNCaP cells; responses that were eliminated when IGF-I was added back to the post-intervention samples. The p53 protein content was increased and NF*κ*B activation reduced in the post serum-stimulated LNCaP cells. Similar results were observed when the IGF-I receptor was blocked in the pre-intervention serum. In androgen-independent PC-3 cells, growth was reduced while none of the other factors were changed by the intervention. We conclude that diet and exercise intervention might help prevent clinical PCa as well as aid in the treatment of PCa during the early stages of development.

## 1. Introduction

Prostate cancer (PCa) is the most common solid-tumor cancer in US men and is the second leading cause of cancer deaths [1]. The incidence of PCa is much higher in US men compared to men in other less-developed countries; however, PCa is on the rise in the less-developed countries as they become more Westernized [2]. These data, in addition to the migration studies [3, 4], strongly suggest that environmental factors play an important role in the development of clinical prostate cancer. Two factors that have received the most attention are dietary fat and physical activity. The international data show that the incidence of PCa correlates with fat consumption; however, cohort studies within a given population have been unable to confirm a significant relationship between dietary fat or fatty food consumption and prostate cancer risk [5]. Epidemiological studies relating physical activity to the risk for PCa have also provided conflicting results.

Thune and Furberg [6] concluded from the 28 studies reviewed that either occupational or leisure physical activity might reduce PCa risk by 10–70%. Lee [7] concluded from a review of 36 studies that the data were inconsistent. It should be noted that countries consuming low levels of dietary fat are also generally more physically active.

We developed a bioassay using cell cultures to investigate the effects of lifestyle change on serum-stimulated growth and apoptosis of PCa cell lines and reported that adopting a low-fat, high-fiber diet of natural foods, combined with daily exercise altered serum factors that reduced growth of androgen-dependent LNCaP and LAPC-4 cell lines; little effect was noted with androgen-independent PC-3 cells [8, 9]. More important than the reduction in growth in the androgen-dependent cell lines, was the observation of increased apoptosis [8]. In response to the diet and exercise intervention we noted reductions in serum free testosterone, estradiol and insulin. When these three hormones were added back to the post diet and exercise serum we could account for less than half of the reduction in cell growth and concluded that other factors must also be changing in response to the diet and exercise that were involved in reducing the growth of the androgen-dependent cell lines [10]. In the study where we found that the diet and exercise intervention induced apoptosis in LNCaP cells we also reported a reduction in serum insulin-like growth factor-I (IGF-I) and an increase in insulin-like growth factor binding protein-1 (IGFBP-1) [8]. The purpose of the present study was to further investigate the mechanisms by which a low-fat, high-fiber diet and exercise intervention might affect growth and apoptosis of PCa cell lines.

## 2. Materials and Methods

### 2.1. Subjects and Intervention

The subjects of this study were five men in their early sixties with no signs of PCa (PSA < 4.0) who attended the Pritikin Longevity Center 3-Week Residential Program in Aventura, FL. During their stay at the Center the men were given prepared meals with 12–15% fat calories, 15–20% protein calories and the majority of calories (65–70%) from unrefined complex carbohydrates high in fiber (>40 g/day). The man ate ad libitum except for animal protein that was limited to 3.5 oz of fish/fowl served 3 days/week and small amounts in soups or casseroles 2 days/week. The men attended daily, supervised exercise classes for 60 min. Details of the program have been previously published [11]. Serum samples were obtained from the men at the start and after 21 days at the center. The serum was frozen and shipped to UCLA on dry ice and was stored at −80°C until analyzed. The study was approved by the Human Subjects Protection Committee at the University of California, Los Angeles and informed consent was obtained from the men.

### 2.2. Cell Cultures

LNCaP and PC-3 prostate cancer cell lines were purchased from American Type Culture Collection (ATCC, Manassa, VA) and grown in 75 cm^2^ flasks in RPMI-1640 medium, 37°C and 5% CO_2_ as described in detail previously [8, 12]. For the growth assays cells were plated (5 × 10^3^ cells/well) in 96-well plates and allowed to attach overnight in 10% fetal bovine serum (FBS). The following day the medium was removed and replaced with fresh 10% FBS (control) or 10% subject serum in duplicate. Growth was determined after 48 h by the CellTiter 96AQ assay (Promega, Madison, WI). For apoptosis 10 × 10^3^ cells/well were plated in the 96-well plates and treated as in the growth assays. Apoptosis was determined by Cell Death Detection ELISA Plus (Roche, Applied Science, Indianapolis, IN) and the results expressed in micro units of mono and oligonucleosomes.

To assess the role of the IGF-I receptor pathway in the response to diet and exercise intervention, *α*IR3 was added to the pre and post serum samples at a concentration of 1 *μ*g/mL prior to measuring growth and apoptosis. To block the phosphatidylinositol 3-kinase (PI3K) pathway LY294002 was added to the pre and post samples at a concentration of 2.8 *μ*M and to block the p38 mitogen-activated protein kinase (MAPK) pathway SB202190 was added at a concentration of 200 nM. The concentrations used were twice the IC_50_ concentrations reported in the literature.

For the IGF-I add back experiments with LNCaP cells, 60 ng/mL of IGF-I was added to the post diet and exercise serum and growth as well as apoptosis were assessed. This is the amount of IGF-I we previously reported to be reduced by the diet and exercise intervention [8].

### 2.3. p53 Protein

In order to assess possible changes in p53 protein, LNCaP and PC-3 cells were plated at a density of 150 × 10^3^ cells/well in 10 cm dishes and allowed to attach and stabilize for 24 h as in the growth and apoptosis assays. The media was then replaced with fresh media containing 10% pre- or post-intervention serum and the cells incubated for 48 h. The cells were then washed with phosphate buffered saline and lysed with 5x Passive Lysis Buffer (Promega) that contained protease inhibitors. After 30 min incubation at room temperature the plates were centrifuged and the supernatants collected. Protein was measured by the bicinchonic acid protein assay (Pierce, Rockford, IL) and p53 determined by ELISA (Calbiochem, SanDiego, CA).

### 2.4. NF*κ*B

To assess activation of NF*κ*B, LNCaP and PC-3 cells were seeded in 96-well plates and after 24 h the medium changed to fresh medium with 10% pre- or post-intervention serum. After 48 h of incubation, phosphorylated and total NF*κ*B were determined using a CASE ELISA kit (Superarray, Frederick, MD). The optical density readings were used to produce a ratio of activated to total NF*κ*B. The ratio is greater than 1.0 due to the fact that the antibody for the phosphorylated NF*κ*B gives a stronger signal than the antibody for total NF*κ*B.

### 2.5. Statistical Analysis

All assays were run in duplicate and the mean obtained for statistical purposes. Statistical analyses were performed by a paired Student's *t*-test using InStat Statistical Software (Graphpad Prizm, SanDiego, CA). Data are expressed as means ± standard error with *P* <  .05 considered significant.

## 3. Results

### 3.1. LNCaP Proliferation

**Figure 1:**
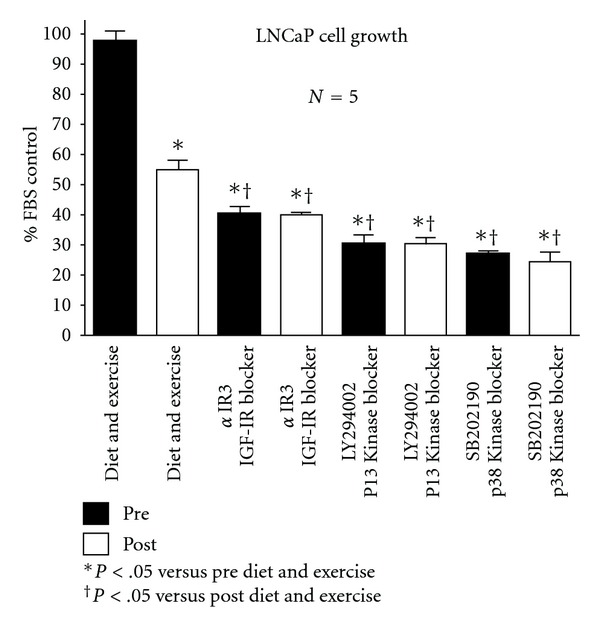
Effect of diet and exercise intervention, as well as various blockers, on the growth of serum-stimulated, androgen-dependent LNCaP cells.

Figure 1 shows the growth data for the LNCaP cells under the various experimental conditions. In response to the diet and exercise intervention, growth of the androgen-dependent LNCaP cells over 48 h was significantly reduced by 44%. When the IGF-I receptor blocker, *α*IR3, was added to the pre and post diet and exercise serum, growth was reduced to the same level and was significantly below the post diet and exercise samples. When the PI3 Kinase blocker, LY294002, was added to the pre and post diet and exercise samples, growth was reduced to the same level and was significantly lower than the tests run with *α*IR3. When the p38 MAP Kinase blocker, SB202190, was added to the pre- and post-diet and exercise samples, growth was again reduced to the same level and was significantly below the *α*IR3 results but not different from the PI3 Kinase blocker results.

### 3.2. LNCaP Apoptosis

**Figure 2:**
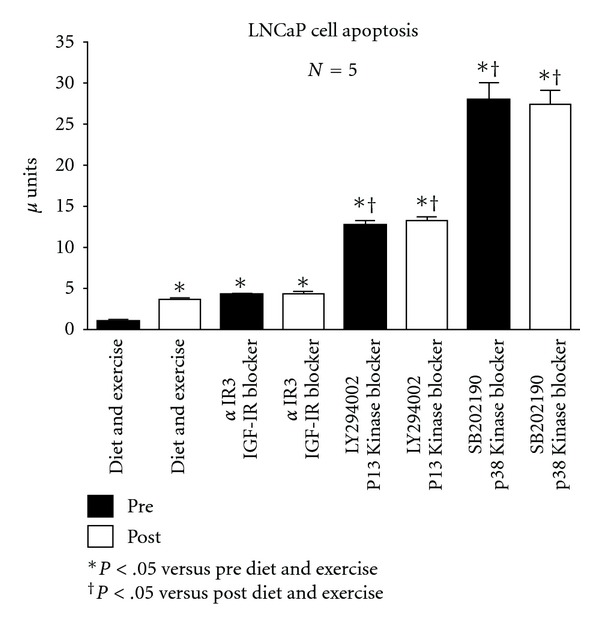
Effect of diet and exercise intervention, as well as various blockers, on apoptosis of serum-stimulated, androgen-dependent LNCaP cells.

Figure 2 shows the apoptosis data for the LNCaP tests run under the various experimental conditions. In response to diet and exercise intervention, apoptosis was increased by 354%. When *α*IR3 was added to the serum, apoptosis was increased by 417% and 424% for the pre- and post-samples that was significantly more that the increase achieved with diet and exercise alone. When the PI3 Kinase blocker, LY294002, was added to the pre and post diet and exercise samples, apoptosis was increased by 1258% and 1306% over the pre samples; compared to the post samples the increases were 252% and 269%. When the p38 MAP Kinase blocker, SB202190, was added to the pre and post diet and exercise samples, apoptosis was further increased to the same level. Compared to the pre samples the increase was 2749% and compared to the post samples was 676%.

### 3.3. PC-3 Proliferation

**Figure 3:**
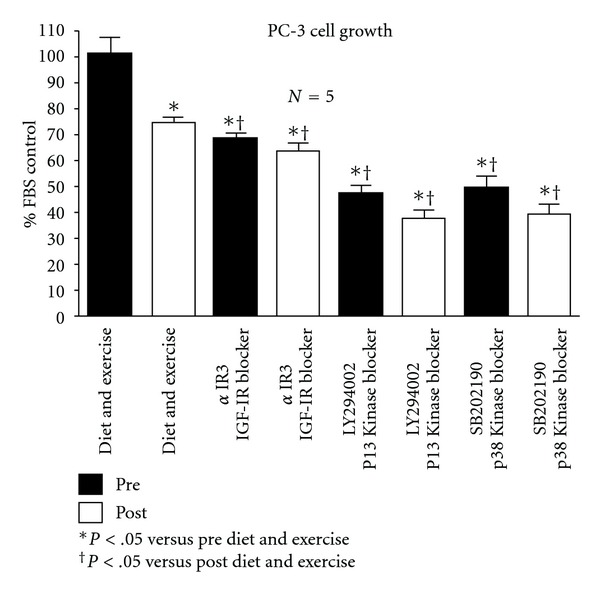
Effect of diet and exercise intervention, as well as various blockers, on the growth of serum-stimulated, androgen-independent PC-3 cells.

Figure 3 shows the growth data for the androgen-independent PC-3 cells under the various test conditions. In response to diet and exercise intervention growth in the PC-3 cells was only reduced by 26.2%, considerably less than the response observed in the LNCaP cells. When *α*IR3 was added to the pre and post diet and exercise serum, growth was reduced to the same level and was a small, but significant reduction compared to the post samples. When the PI3 Kinase blocker, LY294002, was added to the pre and post diet and exercise samples, growth was further reduced to the same level and was more significant than the reduction observed with the diet and exercise intervention as well as the intervention plus *α*IR3. When the p38 MAP Kinase blocker, SB202190, was added to the pre and post diet and exercise samples, the response was very similar to the response observed with the PI3 Kinase blocker.

### 3.4. PC-3 Apoptosis

**Figure 4:**
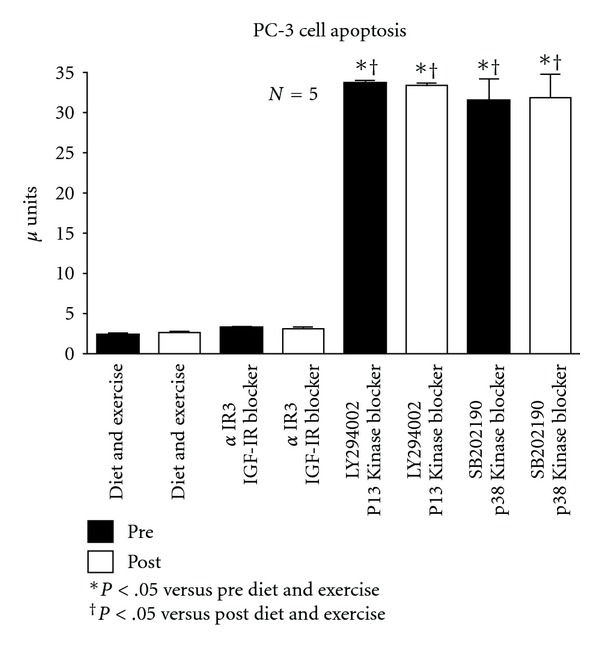
Effect of diet and exercise intervention, as well as various blockers, on apoptosis of serum-stimulated, androgen-independent PC-3 cells.

Figure 4 shows the apoptosis results for the PC-3 cells. Unlike the response in LNCaP cells, there was no increase in apoptosis with diet and exercise intervention or the addition of *α*IR3. The addition of LY294002 or SB202190 resulted in a dramatic increase in apoptosis with no difference between the pre or post intervention samples.

### 3.5. IGF-I Add Back

**Figure 5:**
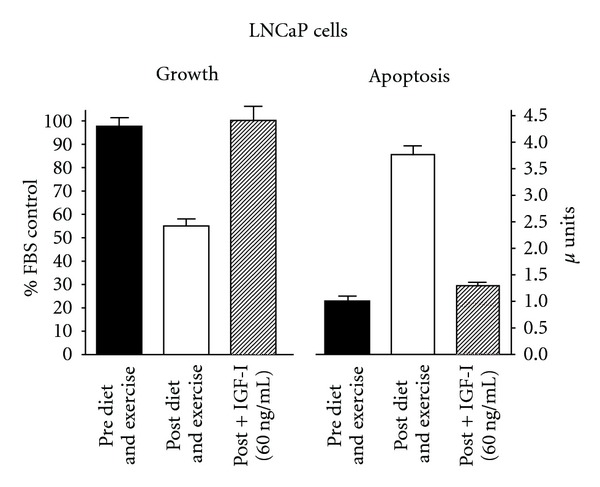
Effect of adding back IGF-I to the post diet and exercise serum on growth and apoptosis of LNCaP cells.

To further investigate the role of reducing IGF-I through diet and exercise intervention, IFG-I was added back to the post diet and exercise intervention serum at 60 ng mL^−1^, the amount we previously found to be reduced with diet and exercise [8]. As can be seen in Figure 5, the addition of IGF-I completely eliminated the reduction in growth and increase in apoptosis observed in LNCaP cells with diet and exercise intervention. As there was no increase in apoptosis in the PC-3 cells with diet and exercise intervention we did not do the add-back experiments.

### 3.6. p53 Protein

The p53 protein content in LNCaP lysates was increased significantly from 47.23 ± 2.12 pg *μ*g^−1^ protein to 67.16 ± 3.2 pg *μ*g^−1^ protein with diet and exercise intervention. When *α*IR3 was added to the pre samples, p53 increased to 73.79 ± 3.4 pg *μ*g^−1^ protein. Compared to the LNCaP cells the p53 protein content in the PC-3 cells was lower, 32.36 ± 3.0 pg *μ*g^−1^ protein, and did not increase significantly with diet and exercise intervention, 38.83 ± 3.0 pg/*μ*g protein, or with the addition of *α*IR3 to the pre serum, 37.07 ± 3.3 pg *μ*g^−1^ protein.

### 3.7. NF*κ*B Activation

In the LNCaP cells the ratio phosphorylated/total NF*κ*B was significantly reduced after 48 hr of growth from 3.0 ± 0.33 to 2.1 ± 0.23 with diet and exercise intervention. In the PC-3 cells the ratio was 1.9 ± 0.02 for the pre samples and was unchanged with diet and exercise intervention, 1.9 ± 0.06.

## 4. Discussion

The pathologic incidence of small, latent or sub-clinical prostate carcinoma is similar across many different populations while the incidence of clinical PCa varies greatly and has been suggested to be dependent on lifestyle [2, 13, 14]. The results from the present study confirm our earlier studies [8, 9] showing that a low-fat, high-fiber diet and daily exercise significantly reduces growth and induces apoptosis in serum-stimulated, androgen-dependent LNCaP cells, indicating that lifestyle might be an important factor in the development of clinical PCa. PCa is thought to originate with the invasion of inflammatory cells resulting in proliferative inflammatory atrophy [15]. Factors responsible for the progression of the disease from the initial stage to prostatic intraepithelial neoplasm and eventually to cancer are not known. It is known that inflammation plays a role not only in the early initiation of the disease but also in the progression of PCa [16]. In a recent study Zhu et al. [17] reported that in resected PCa tumors there was much greater macrophage infiltration into cancer-containing regions compared to normal regions indicating enhanced inflammation. Not only do monocyte/macrophages contain the NF*κ*B inflammatory pathway so do adipocytes and tumor cells. Nguyen et al. [18, 19] reported that fatty acids increase inflammatory cytokine production in both monocytes and adipocytes by activating toll-like receptors 2 and 4. Prostate, as well as other cancer cell lines, also contain toll-like receptors 2 and 4 and when activated increase cell proliferation and resistance to apoptosis [20]. Huang et al. [20] describe the situation as “a double-edged sword". Toll-like receptors are centrally involved in the initiation of the innate and adaptive immune responses via lymphocytes; however, in tumor cells the toll-like receptors activate proinflammatory factors and immunosuppressive molecules that lead to resistance of tumor cells to cytotoxic lymphocyte attack resulting in immune evasion. In the present study we found that NF*κ*B activation was reduced in the androgen-dependent LNCaP cells following the diet and exercise intervention, indicating reduced tumor cell inflammation. The reduction in activation of NF*κ*B in the LNCap cells could have been due to the major reduction in serum lipids previously reported for the intervention as well as an alteration in the ratio of dietary *n*-6 to *n*-3 fatty acids [8–12]. In an earlier study with LAPC-4 xenografts we found that balancing the common *n*-6 fatty acid content of the diet with *n*-3 fatty acid, at the same percent of fat calories, reduced tumor growth that was associated with reduced inflammation as indicated by a reduction in xenograpft cyclooxygenase-2 mRNA and protein [21]. In the typical US diet the ratio of *n*-6 to *n*-3 fatty acid is 10–25 : 1 while the diet used in the present study had a ratio of 2–4 : 1 [22]. Thus, it appears that dietary factors ie. reduction in total fat and the *n*- 6 : *n*-3 fatty acid ratio can influence prostate tumor inflammation, growth and apoptosis.

Another factor that can influence inflammation, as well as proliferation and apoptosis is activation of the PI3 kinase/Akt pathway [23]. Akt causes the NF*κ*B inhibitory binding protein IkB to release NF*κ*B that is then translocated to the nucleus where it transcribes multiple genes involved in inflammation, proliferation, and antiapoptosis. Several factors including growth factors, cytokines and chemokines have been shown to activate the PI3 kinase/Akt pathway. The MAPK pathway has also been shown to activate NF*κ*B and increase cytokine production [24]. Our study focused on IGF-I as it activates both of these pathways. We previously reported that serum IGF-I was decreased while IGFBP-1 was increased in response to the diet and exercise intervention [8]. We thus blocked the IGF receptor with *α*IR3 and found that growth was reduced to the same level in the pre- and post-diet and exercise samples, a little below the level observed with diet and exercise alone. These data are interpreted as indicating that the reduction in growth achieved with diet and exercise is due primarily to a reduction in IGF-I activity. Apoptosis was significantly increased in LNCaP cells with diet and exercise intervention in agreement with our earlier report [8]. Apoptosis was increased with *α*IR3 to the same level for the pre and post samples and slightly higher than the level achieved with diet and exercise intervention. We then added IGF-I back to the post diet and exercise intervention serum and completely reversed the decrease in growth and increase in apoptosis in the LNCaP cells. The results from these two experiments were interpreted as indicating that the decrease in growth and increase in apoptosis in serum-stimulated LNCaP cells with diet and exercise intervention were due primarily to a reduction in serum IGF-I.

To further investigate the role of the IGF-I receptor in the response to diet and exercise intervention, we employed LY29400, a PI3 kinase inhibitor, and SB202190, a p38 MAPK inhibitor. Both pathways are activated by IGF-I. When the PI3 kinase inhibitor was added to the pre and post samples, growth of the LNCaP cells was reduced to the same level and slightly below the growth reduction seen with the *α*IR3. Apoptosis, however, was greatly increased compared to the level seen with the diet and exercise or when the *α*IR3 was used to block the IGF-I receptor. The increase in apoptosis was greater with the p38 MAPK inhibitor compared to the PI3K inhibitor. These results indicate that factors in the serum, other than IGF-I, stimulate growth and especially inhibit apoptosis via the PI3K and p38 MAPK pathways. What exactly the additional factors are is not known but they appear not to respond to diet and exercise intervention.

To further understand how the diet and exercise intervention was working to reduce growth and induce apoptosis in the LNCaP cells, p53 protein was measured in the lysates. We hypothesized that p53 protein would increase with diet and exercise intervention based on reports from Heron-Milhavet et al. [25, 26] showing that when IGF-I was added to DNA-damaged cells p53 protein was reduced as a result of Mdm-2 ubiquitination, as opposed to being increased in response to the DNA damage. Our hypothesis was correct as p53 protein did increase in response to the diet and exercise intervention as well as when *α*IR3 was added to the pre intervention samples. The increase in p53 protein could be a major factor in the reduced growth and increased apoptosis seen with diet and exercise as p53 is known to activate p21, a cyclin-dependent kinase inhibitor [27]. It also stimulates the caspase system to increase apoptosis [27]. These results agree with our data on exercise showing an increase in p53 protein in serum-stimulated LNCaP cells [12]. In the typical adult male serum (pre-samples) factors other than IGF-I that are not reduced with diet and exercise must be stimulating growth and inhibiting apoptosis via the PI3K and MAPK pathways as indicated by the results obtained with their specific inhibitors.

Results with the androgen-independent PC-3 cells were far less impressive. Diet and exercise intervention reduced growth, but less than in the LNCaP cells, and did not increase apoptosis. Furthermore, there was no decrease in NF*κ*B activation or increase in p53 protein with the diet and exercise intervention. The fact that p53 protein did not increase is not surprising as it is well known that the PC-3 cell line has a defective p53 gene, similar to many advanced cancers [28, 29]. Conversely, the LNCaP cell line has an intact p53 gene but the response of the gene appears to be suppressed by the high levels of IGF-I found in the typical male serum.

The results from this study using the two cell lines, androgen-dependent LNCaP and androgen-independent PC-3, suggest that a low-fat, high-fiber diet and daily exercise might be effective for halting or slowing the progression of early stage PCa but would be of lesser value for end-stage PCa. In a randomized clinical trial Ornish et al. [30] reported that men with early-stage PCa placed on “watchful waiting” who were treated with a low-fat, vegetarian diet, regular exercise, and stress management had serum changes that reduced the growth of serum-stimulated LNCaP cells similar to the present study. At one year of follow-up, 6 of the 43 control patients versus 0 of 41 diet and exercise patients required treatment due to rising prostate specific antigen or advancing PCa as determined by their physician. In a recent gene-chip study with biopsy tissue Ornish etal. [31] reported changes in prostate tissue 3 months after the intervention including up-regulation of 48 genes and down-regulation of 453 genes, many related to PCa. These results clearly demonstrate the diet and exercise intervention can impact prostate tissue.

## 5. Conclusions

A low-fat, high-fiber diet and daily exercise intervention altered serum factors that reduced growth and increased apoptosis in serum-stimulated, androgen-dependent LNCaP cells. In androgen-independent PC-3 cells the intervention reduced growth, less than in LNCaP, and did not increase apoptosis. Adding IGF-I back to the post-intervention serum blocked the responses in LNCaP cells. The p53 protein content was increased in LNCaP cells with the intervention but not in the PC-3 cells. When *α*IR3 was added to the pre-samples to block IGF-I, p53 protein also increased. NF*κ*B activation was decreased in the LNCaP cells with the intervention, but was unchanged in the PC-3 cells. Unfortunately we ran out of serum and were unable to complete the proposed studies with *α*IR3 to document the involvement of IGF-I in NF*κ*B activation in the LNCaP cells. This is an important limitation to the study.

**Figure 6:**
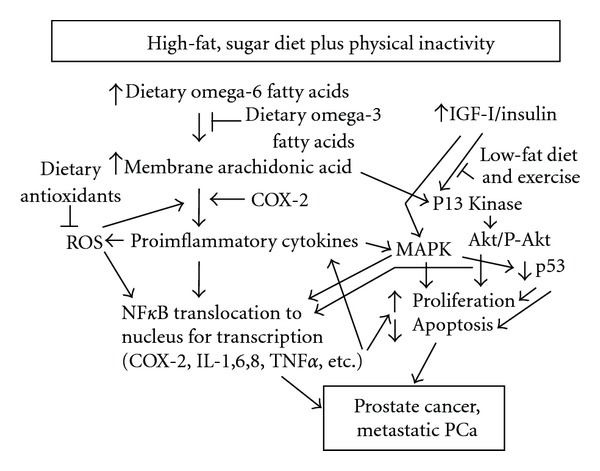
A hypothetical model to explain how adopting a low-fat, high-fiber diet and daily exercise might reduce the risk for PCa by reducing prostate inflammation and IGF-I/insulin signaling, IGF-I, insulin-like growth factor-I; PI3 kinase, phosphatidylinositol-3 kinase; Akt, protein kinase B; MAPK, mitogen-activated protein kinase (p38); COX-2, cyclooxygenase-2; ROS, reactive oxygen species; IL-1, interlukin-1, TNF*α*, tumor necrosis factor-alpha.

Based on the results of this study, in addition to data from the literature, Figure 6 presents a hypothetical description of how the typical US lifestyle (high-fat, refined-sugar diet and physical inactivity) increases the risk for PCa via inflammatory and IGF-I/insulin pathways. Adopting a low-fat, high-fiber diet and daily exercise might reduce the risk for the development of clinical PCa by reducing inflammation and IGF-I/insulin signaling. This lifestyle change might also aid in the treatment of early-stage cancer, but would be of lesser benefit for late-stage, androgen-independent PCa where p53 defects are likely.

## Funding

National Cancer Institute (NCI) Specialized Program of Research Excellence Grant P50 CA-921310, NCI Grant R01 CA-100938; donation from the L.B. Research and Education Foundation. Dr Barnard has declared a conflict of interest as he is a consultant to the Pritikin Longevity Center where the serum samples were obtained.
